# 2014: Identification of novel shared tumor antigens for the development of T-cell-based immunotherapies

**DOI:** 10.1017/cts.2017.194

**Published:** 2018-05-10

**Authors:** Sherille Bradley, Greg Lizee

## Abstract

OBJECTIVES/SPECIFIC AIMS: The specific objective of this proposal is to identify and validate targetable tumor-associated antigens (TAAs) in ovarian and pancreatic cancer. It is our central hypothesis that the accurate identification and selection of appropriate TAAs will provide a foundation on which to develop of novel and effective cancer immunotherapies. We have formulated this hypothesis on the basis of preliminary results in which we have used high-throughput tandem mass spectrometry (MS) to successfully identify TAAs from melanoma patient tumors. We have subsequently generated TAA-specific T-cells that showed specific recognition and killing of tumor cells, and will form the basis of an upcoming clinical trial for our melanoma patients. We now have extended this antigen identification pipeline into ovarian cancer to accomplish our objective of developing effective T-cell-based immunotherapies for ovarian cancer and pancreatic patients. METHODS/STUDY POPULATION: We have collected patient tumor specimens, and we performed HLA immunoprecipitation, peptide elution, and completed high-throughput tandem MS on these eluted samples to identify TAAs. In addition, we have validated the safety of potential targets through the use of the publicly available RNA sequence data sources GTEX and TCGA. RESULTS/ANTICIPATED RESULTS: To date, we have successfully completed over 60 peptide elutions from ovarian and pancreatic patients samples. In total, we have found several potential novel tumor-associated targets. VGLL1, is one of these identified antigens, and in conjunction with our collaborators, we have successfully generated T-cells against it. Additionally, we have found that VGLL1 is a potential novel TAA for 3 other cancer types, including bladder, gastric, and triple negative breast cancers. We are now focusing our efforts on testing these T-cells against additional ovarian cancer cell lines and these cancer types to determine their specificity. We plan to continue the generation and testing other identified potential TAAs as well. We plan to use these T-cells directly in clinical trials in the future. DISCUSSION/SIGNIFICANCE OF IMPACT: The rationale for this proposal is that through the identification and validation of TAAs, we can open the door to a new world of therapies that can potentially increase the survival rate in a disease with a historically grim prognosis.

